# POEMS (Polyneuropathy, Organomegaly, Endocrinopathy, Monoclonal Plasma Cell Disorder, and Skin Changes) Syndrome as a Sequela of Castleman Disease: A Case Report

**DOI:** 10.7759/cureus.49330

**Published:** 2023-11-24

**Authors:** Syed Abdullah Haider, Sadia Iram, Asma Abdul Rashid, Anusha Manazar, Hamza Javed

**Affiliations:** 1 Medicine, University College of Medicine and Dentistry, Lahore, PAK; 2 Medicine, Lahore Medical and Dental College, Lahore, PAK; 3 Oncology, University of Lahore Teaching Hospital, Lahore, PAK

**Keywords:** polyneuropathy, autologous stem cell transplantation, claw hand, hdt, castleman variant of poems syndrome

## Abstract

Polyneuropathy, organomegaly, endocrinopathy, monoclonal plasma cell disorder, and skin changes (POEMS) syndrome is a rare multisystemic paraneoplastic disorder caused by an underlying plasma cell dyscrasia. Its diagnosis is based on the presence of two mandatory criteria and at least one major and one minor criterion. We report a case of a 52-year-old female patient who presented with complaints of acrocyanosis, night sweats, scaly skin, and swelling on the left side of the neck. She was a known case of hypothyroidism, antiphospholipid syndrome, and cerebral venous thrombosis, and had other comorbidities as well. She also exhibited weakness and paresthesia of the limbs and muscle wasting in the hands. All necessary examinations and investigations were performed and the patient was eventually diagnosed with POEMS syndrome. She underwent chemotherapy along with immunotherapy initially, but as the disease relapsed, she was referred for high-dose therapy (HDT) and autologous stem cell transplantation.

## Introduction

Polyneuropathy, organomegaly, endocrinopathy, monoclonal plasma cell disorder, and skin changes (POEMS) syndrome is a multisystemic disorder that occurs due to plasma cell dyscrasia. It was described by Crow in 1956, then Fukase in 1968, and the acronym "POEMS" was coined by Bardwick et al. in 1980 [1]. Its signs and symptoms include endocrine disorders, sclerotic bone lesions, monoclonal plasma cell proliferative disorder, progressive sensorimotor polyneuropathy, organ enlargement, lymph node hyperplasia, elevated vascular endothelial growth factor (VEGF) levels, edema, ascites, pleural effusion, darkening of the skin (hyperpigmentation) and papilledema [2]. POEMS syndrome is associated with a rise in pro-inflammatory cytokines. The severity of the disease activity correlates with VEGF levels [3]. The diagnosis is based on certain major and minor criteria (discussed later in this study) and can often be missed due to the similarity of signs and symptoms with other disorders. The median survival rate is 8-14 years without treatment [4].

## Case presentation

Case history

A 52-year-old female patient presented to the emergency department of the University of Lahore Teaching Hospital, Lahore, Pakistan with a constellation of symptoms, including night sweats, scaly skin, rash, acrocyanosis, and multiple swellings on the left side of her neck which she had been experiencing for three to four months prior to her presentation. These neck swellings were progressively increasing in size and were characterized as mobile, non-tender, and firm. The patient's medical history was remarkable for hypothyroidism, antiphospholipid syndrome, and cerebral venous thrombosis, which she had been living with for the past six years. Subsequently, she developed hypertension and diabetes mellitus a year and a half ago. She also exhibited wasting and deformity of the hands, weakness in the proximal muscles of the upper limbs, and weakness and paresthesia in the distal portions of both lower limbs, gradually progressing over a period of six months.

Physical examination

**Figure 1:**
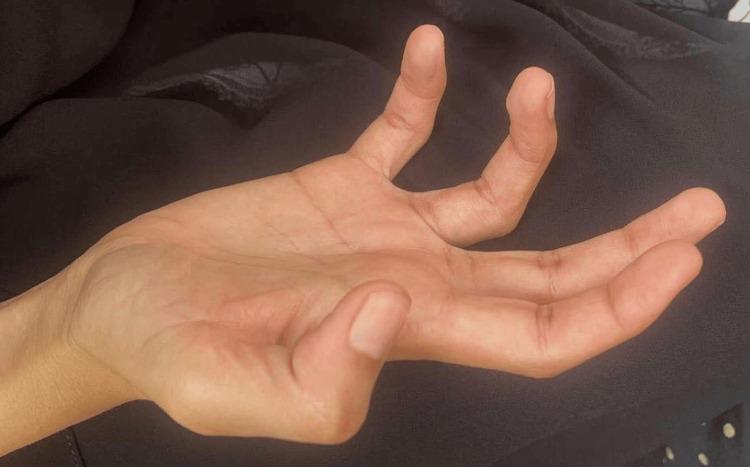
Claw hand deformity in the right hand of the patient along with muscle wasting as a sequela of neuropathy

Physical examination revealed desquamated skin rashes with multiple skin lesions. An examination of the neck revealed enlarged left supraclavicular, anterior cervical, left cervical, right axillary, bilateral occipital, and submandibular lymph nodes. Abdominal examination showed an enlarged liver (20 cm span) with signs of ascites and a protuberant stomach. Ophthalmic examination confirmed papilledema and neurologic evaluation revealed mild paresthesia and tetraparesis. Bilateral claw hands were a notable clinical feature (Figure 1).

Investigations

**Figure 2:**
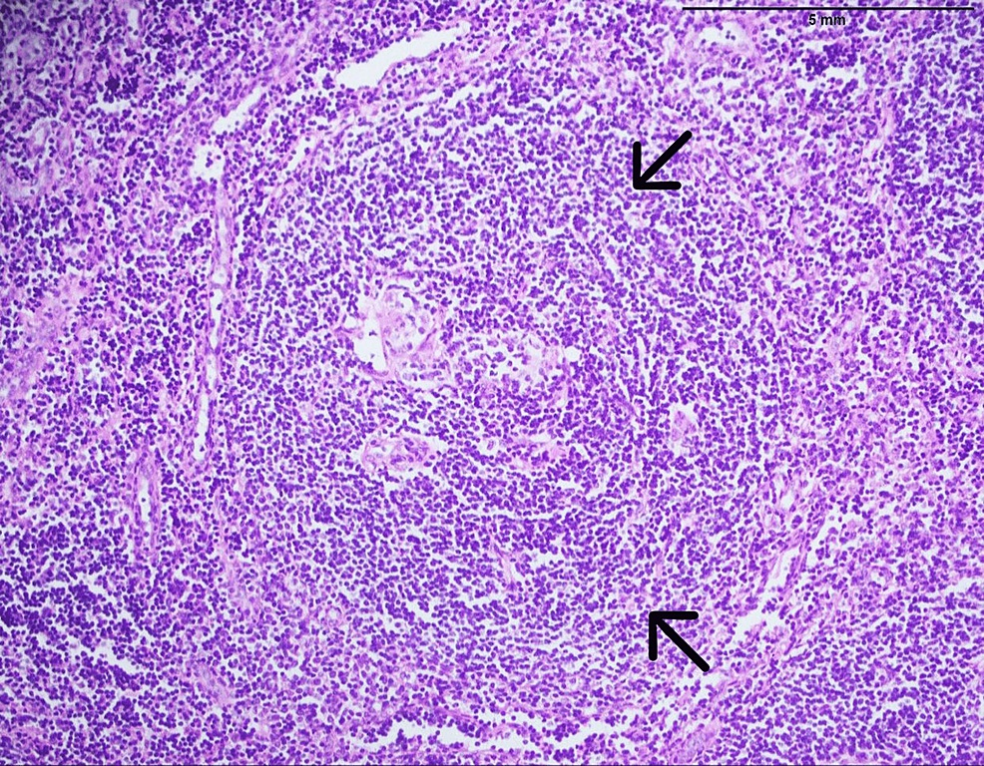
H & E staining of cervical lymph node biopsy (magnified 20x) The image shows the proliferation of plasmacytoid cells with eccentric nuclei. The mantle zone shows an onion skin appearance with some follicles showing more than one germinal center

Laboratory investigations showed a T3 level of 46.6 ng/dl (reference range: 84-172 ng/dl), T4 level of 3.79 mcg/dl (reference range: 4.5-12.5 mcg/dl), and a TSH level of 12.1 mIU/L (reference range: 0.4-4 mIU/L); HbA1c and plasma glucose levels indicated the presence of diabetes mellitus. Serum protein electrophoresis confirmed the presence of IgG lambda monoclonal protein. Ultrasound of the neck revealed an enlarged right lobe of the thyroid gland (measuring 44 x 22 x 19 mm) with a 5-mm cyst. Contrast-enhanced CT scans of the neck, chest, abdomen, and pelvis revealed moderately large left cervical lymph nodes, with one measuring up to 38 mm, an enlarged right axillary lymph node of 15.5 mm, minor atelectasis in the lungs, and mild hepatosplenomegaly. A biopsy of the left cervical lymph node was done, which confirmed the diagnosis of Castleman disease, the hyaline vascular type, without evidence of malignancy (Figure 2). Nerve conduction studies revealed polyneuropathy in all limbs (nerve conduction velocity: <33 m/s). An FDG PET-CT scan indicated sclerotic foci in some thoracic and lumbar vertebrae along with bilateral pleural and pericardial effusion. The diagnosis of POEMS syndrome was established based on the fulfillment of diagnostic criteria, including the presence of polyneuropathy and monoclonal plasma proteins (lambda) as mandatory criteria, Castleman disease and sclerotic bone lesions as major criteria, and organomegaly, skin changes, edema, papilledema, and endocrinopathies as minor criteria.

Treatment

The patient received high-dose alkylator-based chemotherapy in combination with immunotherapy. Following the completion of the treatment course, there was a notable improvement in symptoms, including a reduction in the size of neck swellings, decreased bone pain, and improved frozen shoulder. Subsequent FDG PET-CT scans indicated disease stability in the neck and axilla with mild avid. However, the patient later presented with worsening symptoms, indicating disease relapse. She underwent another round of chemotherapy, which provided temporary relief. A follow-up FDG PET-CT scan after six months of treatment revealed the presence of significantly enlarged cervical and right axillary lymph nodes non-avid, bilateral metabolically inactive pleural and pericardial effusion, and worsening endocrinopathy on blood reports. The patient was administered symptomatic treatment and referred to the transplant unit for high-dose therapy (HDT) with autologous stem cell transplantation.

## Discussion

POEMS syndrome, also called Crow-Fukase syndrome or Takatsuki syndrome, is a rare multisystemic disorder caused by an underlying plasma cell neoplasm. The acronym POEMS stands for polyneuropathy, organomegaly, endocrinopathy, monoclonal plasma cell disorder, and skin changes [5].

The diagnosis involves fulfilling two mandatory criteria: polyneuropathy (demyelinating) and monoclonal plasma cell proliferative disorder; one out of the following three major criteria: elevated vascular endothelial growth factor (VEGF), sclerotic bone lesions, and Castleman disease; and one of the following six minor criteria: organomegaly, endocrinopathy, extravascular volume overload, skin changes, papilledema, and thrombocytosis/polycythemia [6]. The diagnostic criteria are elaborated in Table 1 [2]. Castleman disease is a lymphoproliferative disorder characterized by an increase in inflammatory cytokines leading to B cell proliferation, and it affects lymph nodes of the body. Due to its striking resemblances with myeloma or monoclonal gammopathy of undetermined significance (MGUS), these patients are sometimes misdiagnosed [7]. The condition may take years to develop fully and patients suffer in the absence of a definitive diagnosis, predominantly due to varying clinical presentations [8].

**Table 1:** Criteria for the diagnosis of POEMS syndrome* *[9] The diagnosis of POEMS syndrome is confirmed when both the mandatory major criteria, 1 of the 3 major criteria, and 1 of the 6 minor criteria are present ^a ^There is a Castleman disease variant of POEMS syndrome that occurs without evidence of a clonal plasma cell disorder that is not accounted for in this table. This entity should be considered separately ^b ^Because of the high prevalence of diabetes mellitus and thyroid abnormalities, this diagnosis alone is not sufficient to meet this minor criterion ^c ^Approximately 50% of the patients have bone marrow changes that distinguish it from a typical monoclonal gammopathy of undetermined significance or myeloma bone marrow. Anemia or thrombocytopenia are distinctly unusual in this syndrome unless Castleman disease is present

Criteria type	Description^a^
Mandatory major criteria	1. Polyneuropathy (typically demyelinating). 2. Monoclonal plasma cell proliferative disorder (almost always lambda)
Other major criteria (1 required)	3. Castleman disease^a^. 4. Sclerotic bone lesions. 5. Increased levels of vascular endothelial growth factor
Minor criteria	6. Organomegaly (splenomegaly, hepatomegaly, or lymphadenopathy). 7. Extravascular volume overload (edema, pleural effusion, or ascites). 8. Endocrinopathy (adrenal, thyroid^b^, pituitary, gonadal, parathyroid, pancreatic^b^). 9. Skin changes (hyperpigmentation, hypertrichosis, glomeruloid hemangiomata, plethora, acrocyanosis, flushing, white nails). 10. Papilledema. 11. Thrombocytosis/polycythemia^c^
Other symptoms and signs	Clubbing, weight loss, hyperhidrosis, pulmonary hypertension/restrictive lung disease, thrombotic diathesis, diarrhea, low vitamin B12 value

According to a study conducted in China involving 1946 cases, the most common first symptom was peripheral neuropathy (60.44%), followed by extravascular volume overload and endocrine abnormalities [5]. Another study analyzed 14 cases, and all of them were found to have peripheral neuropathy with predominant motor weakness, followed by organomegaly (92.9%) and skin hyperpigmentation (78.6%) [10].

Radiography, ultrasonography, CT, and MRI can be used for the diagnosis of bone lesions and organomegaly. Electromyography and nerve conduction tests are often used for the diagnosis of polyneuropathy in which demyelination, axonal degeneration, and weak amplitudes of sensory and motor nerve action potentials can be seen. Serum electrophoresis is used to diagnose plasmacytoma, which shows a low level of monoclonal IgG with a lambda light chain, which is characteristic of POEMS syndrome [11]. CT-guided lymph node biopsy may reveal findings like hyperplasia and granuloma formation [12] and pulmonary symptoms like pleural effusion and pulmonary hypertension may be present as well [13].

The treatment options include radiotherapy, corticosteroids for immunosuppression, autologous stem cell transplant, alkylators, and thalidomide [14]. Systemic therapy should be initiated in patients with sclerotic lesions or bone marrow involvement. For the temporary suppression of the disease, corticosteroids like dexamethasone are used [15].

## Conclusions

POEMS syndrome is a rare multisystemic disorder associated with high morbidity and mortality if left untreated. It is typically seen in elderly individuals. We documented a case of a 52-year-old patient displaying classic POEMS syndrome symptoms. Early diagnosis and prompt treatment are essential to avert irreversible harm. Currently, various treatment modalities are in use worldwide. However, further large-scale clinical trials are necessary to determine the most effective treatment approach.
